# Identification of hub genes and construction of an mRNA-miRNA-lncRNA network of gastric carcinoma using integrated bioinformatics analysis

**DOI:** 10.1371/journal.pone.0261728

**Published:** 2021-12-30

**Authors:** Gang Wei, Youhong Dong, Zhongshi He, Hu Qiu, Yong Wu, Yongshun Chen

**Affiliations:** 1 Department of Clinical Oncology, Renmin Hospital of Wuhan University, Wuhan, China; 2 Department of Clinical Oncology, The First People’s Hospital of Xiangyang, Xiangyang, China; University of Science and Technology Liaoning, CHINA

## Abstract

**Background:**

Gastric carcinoma (GC) is one of the most common cancer globally. Despite its worldwide decline in incidence and mortality over the past decades, gastric cancer still has a poor prognosis. However, the key regulators driving this process and their exact mechanisms have not been thoroughly studied. This study aimed to identify hub genes to improve the prognostic prediction of GC and construct a messenger RNA-microRNA-long non-coding RNA(mRNA-miRNA-lncRNA) regulatory network.

**Methods:**

The GSE66229 dataset, from the Gene Expression Omnibus (GEO) database, and The Cancer Genome Atlas (TCGA) database were used for the bioinformatic analysis. Differential gene expression analysis methods and Weighted Gene Co-expression Network Analysis (WGCNA) were used to identify a common set of differentially co-expressed genes in GC. The genes were validated using samples from TCGA database and further validation using the online tools GEPIA database and Kaplan-Meier(KM) plotter database. Gene set enrichment analysis(GSEA) was used to identify hub genes related to signaling pathways in GC. The RNAInter database and Cytoscape software were used to construct an mRNA-miRNA-lncRNA network.

**Results:**

A total of 12 genes were identified as the common set of differentially co-expressed genes in GC. After verification of these genes, 3 hub genes, namely *CTHRC1*, *FNDC1*, and *INHBA*, were found to be upregulated in tumor and associated with poor GC patient survival. In addition, an mRNA-miRNA-lncRNA regulatory network was established, which included 12 lncRNAs, 5 miRNAs, and the 3 hub genes.

**Conclusions:**

In summary, the identification of these hub genes and the establishment of the mRNA-miRNA-lncRNA regulatory network provide new insights into the underlying mechanisms of gastric carcinogenesis. In addition, the identified hub genes, *CTHRC1*, *FNDC1*, *and INHBA*, may serve as novel prognostic biomarkers and therapeutic targets.

## Introduction

Gastric carcinoma (GC) is a common malignant tumor originating from the gastric mucosal epithelium. Despite its worldwide decline in incidence and mortality rates over the past decades, GC has a poor prognosis [1]. In 2020, there were about 1,089,103 new gastric cancer cases, which resulted in 768,793 deaths, making it the fifth-most commonly diagnosed cancer type and the fourth leading cause of cancer-related deaths after lung, colorectal, and liver cancers [2]. Although the pathogenesis of gastric cancer remains unclear to date, gastric cancer is widely considered to be a highly heterogeneous disease caused by multiple factors, including chronic infection with Helicobacter pylori [3, 4], Epstein–Barr virus [5], unhealthy diet [6], smoking [7], *etc*., which interact with genes and ultimately lead to tumor development [8, 9]. A gene mutation can be an early indicator of the risk of cancer development and even its future aggressiveness, and genes whose expression is correlated with the progression and prognosis of GC need to be identified. In recent years, biomarkers and therapeutic targets for GC have greatly contributed to improving the diagnosis and treatment of GC. For example, IDO1 and COL12A1, which were found to synergistically promote gastric cancer metastasis, appear to be promising targets for the treatment of gastric cancer [10]. Sha *et al*. [11] found that ORAI2 promotes gastric cancer cell migration and tumor metastasis through MAPK-dependent focal adhesion disassembly and PI3K/Akt signaling, which suggests the possibility of developing potential therapies for GC by targeting the ORAI2 signaling pathway. However, identifying novel diagnostic and prognostic biomarkers remains urgently necessary in view of the biological complexity, poor prognosis, and high reoccurrence of GC.

In the past few decades, microarray technology and bioinformatics analysis have been widely used in cancer functional genomics research to identify genes closely related to tumor development, progression and prognosis through genomics and clinical data analysis [12, 13]. Accordingly, an approach integrating technologies is helpful to identify key genes associated with gastric cancer development and progression. In this study, two major transcriptome analysis methods were used to identify GC-associated genes. One method is the analysis of differentially expressed genes (DEGs), which is used to determine quantitative changes in expression levels between different groups [14]. Studies to identify DEGs between groups under specific conditions, which are widely conducted using RNA-seq data analysis, are critical to understanding phenotypic variation. Differential gene expression analysis can provide in-depth insights into the genetic mechanisms of different phenotypes. For example, through the differential gene expression analysis of multiple data sets, a total of 31 hub genes were identified in colorectal cancer, and these hub genes were found to be significantly enriched in multiple pathways, mainly those related to the cell cycle process, and chemokines and G-protein coupled receptors [15]. The other method is Weighted Gene Co-expression Network Analysis (WGCNA) [16], a data mining-based method used to analyze biological networks, which is used to identify highly coordinated gene sets. Then, based on the interconnectivity of the identified gene sets, candidate biomarker genes or therapeutic targets as well as the association between gene sets and phenotypes can be identified. Compared with focusing only on DEGs, WGCNA can be used to analyze thousands, or nearly thousands, of the genes with the most altered expression, as well as the information about all genes, to identify the gene sets of interest and perform significant association analysis between the genes sets and the phenotype. For instance, Yin *et al*. [17] used WGCNA to identify 5 hub genes that may play a key role in the progression of hepatocellular carcinoma. Additional studies using WGCNA have shown that four genes (*RACGAP1*, *ZWINT*, *TKI*, and *LMNB1*) may serve as potential diagnostic and prognostic markers [18]. In this study, WGCNA was based on the correlation of variables to establish a gene interaction network within the biological system, using the transcriptome and clinical data to identify the modules of genes with characteristic co-expression pattern, and further examine the relationship between the gene modules and the clinical traits [19]. Therefore, we used two approaches, combining the results of the WGCNA and differential gene expression analysis, to enhance the identification of highly correlated genes, which could thus serve as candidate biomarkers for GC prognosis.

## Materials and methods

### Data sources and pre-processing

The gene expression profiles in dataset GSE66229, which consists of the GSE62254 and GSE66222 datasets, obtained on the GPL570 platform using the Affymetrix Human Genome U133 Plus 2.0 array, (Affymetrix Inc., Santa Clara, CA, USA), were downloaded from the Gene Expression Omnibus (GEO) database (https://www.ncbi.nlm.nih.gov/gds). The GSE66222 dataset contains data from 100 samples of normal gastric tissue, and the GSE62254 dataset contains data from 300 gastric cancer samples. The probes were converted into gene symbols according to the annotation file provided by the manufacturer, and probes corresponding to multiple genes were removed. If multiple probes corresponded to one gene, the median value was used. Ultimately, a total of 20,549 genes were subjected to further analysis.

The RNA-seq expression profiles (count format) and clinical data of GC patients were obtained from The Cancer Genome Atlas (TCGA) database (https://portal.gdc.cancer.gov/). Genes with an average expression level value below 1 in all samples were removed. The mRNA expression matrix consists of 22,634 genes and 407 samples, of which 375 are tumor samples and 32 are normal tissue adjacent to the tumor. A total of 371 tumor samples were available for survival analysis.

### Weighted gene co-expression network analysis

All data analysis was performed using the R software (Version 3.63, https://www.r-project.org/). The R package WGCNA was used to analyze the gene co-expression network of the two datasets. First, the genes with the absolute median difference in the top 5,000 were retained. Second, the samples were clustered and the outliers were removed. To construct a scale-free network, soft strengths of β = 3 and 4 were chosen for these two datasets with the function pickSoftThreshold, separately. In the co-expression network, genes with high absolute correlation were aggregated into modules with different colors by using the function blockwiseModules. Then, the correlations between modules and clinical feature information were calculated using the WGCNA package, and modules with high correlation with tumor traits were further analyzed.

### Analysis of differentially expressed genes

The R package edgeR was used to identify DEGs in TCGA datasets. For the GEO data, the limma package was used to identify DEGs between the tumor samples and normal samples. False discovery rate (FDR) was used to adjust the P-value. Genes with |FC| (fold change)> = 2 and adjusted P < 0.05 were considered to be DEGs. The DEGs of TCGA datasets and the GSE66229 datasets were visualized as volcano plots using the R package ggplot2.

### Validation of hub genes

In the two datasets, the overlapping genes were selected from upregulated genes in DEGs and the module genes in the co-expression network, which were visualized using the VennDiagram package [20]. To identify the true hub genes, Kaplan-Meier survival analysis was performed to evaluate the association of hub genes with overall survival (OS) in TCGA datasets using the survival package. Tumor samples with follow-up time were divided into two groups according to the median value of gene expression. Genes with p-values < = 0.05 are verified again in two online databases the GEPIA database (http://gepia.cancer-pku.cn/) and Kaplan-Meier (KM) plotter database (https://kmplot.com/analysis/). Then, the Human Protein Atlas (HPA) database (http://www.proteinatlas.org/) was used to validate the hub genes by immunohistochemistry (IHC).

### Gene set enrichment analysis of real hub genes

In TCGA datasets, samples of GC were divided into two groups according to the expression level of the hub genes (high expression *vs*. low expression based on the median expression value of each hub gene). The gene set enrichment analysis (GSEA) software downloaded from http://www.gsea-msigdb.org/gsea/index.jsp was used to identify the potential function of the hub genes. FDR P <0.05 was used as the criterion for significant enrichment.

### Construction of lncRNA-miRNA-hub gene network

RNAInter database (https://www.rna-society.org/rnainter/), a complete resource of RNA interactome data from the literature and other databases containing over 41 million RNA-related interactions of RDI, RCI, RPI, RHI, and RRI [21], was used to investigate the relationship between mRNAs, long non-coding RNAs (lncRNAs) and microRNAs (miRNAs). We used the lncRNA–miRNA and mRNA–miRNA relationships with strong experimental evidence for further analysis. Then, the lncRNA-miRNA-mRNA regulatory network was visualized with the Cytoscape software.

## Results

### Identification of modules associated with tumor and normal tissues

**Fig 1:**
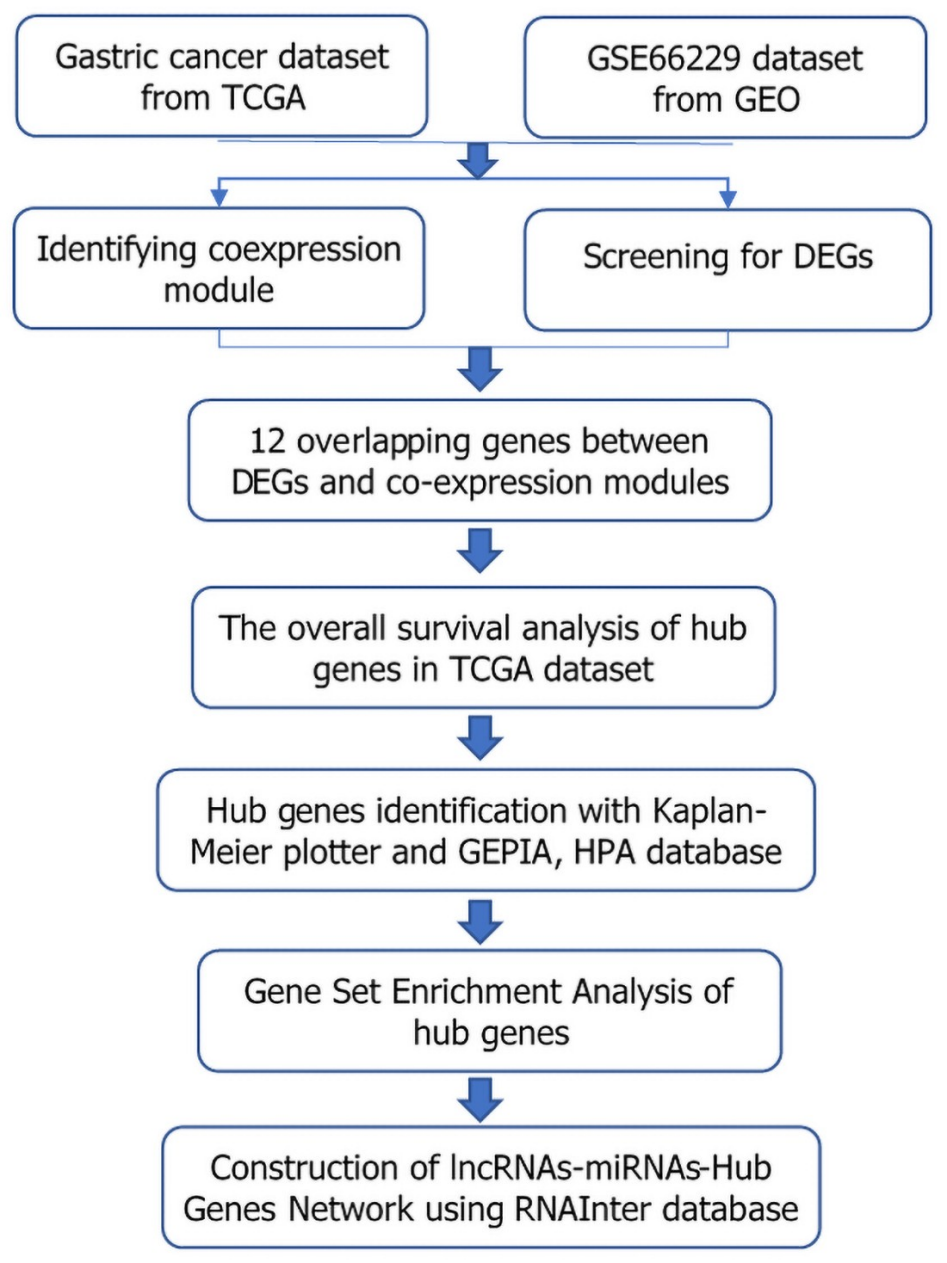
Study design and workflow of this study.

**Fig 2:**
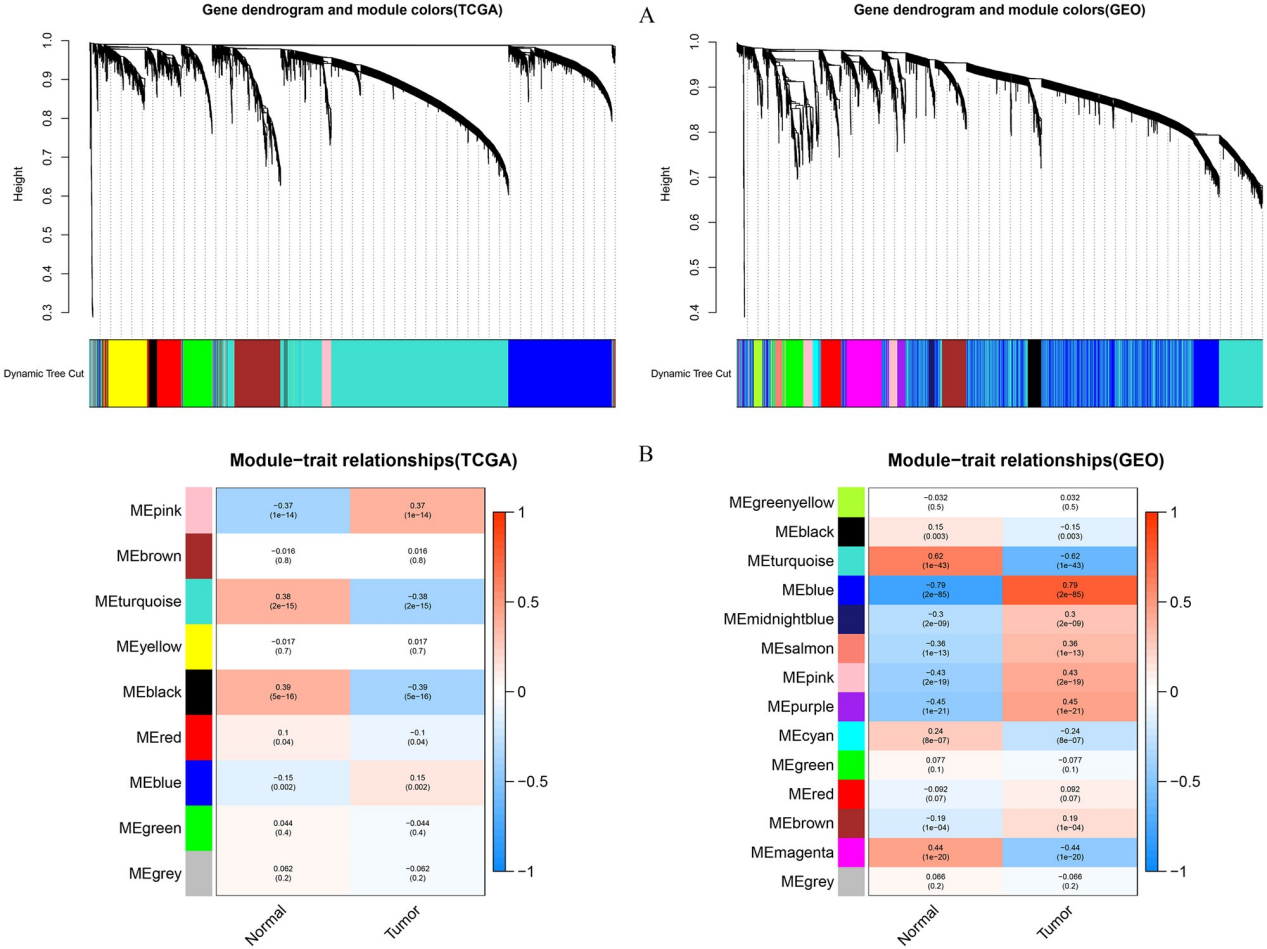
Identification of co-expression modules associated with clinical features in gastric cancer. The results on the left are from TCGA, and those on the right are from GSE66229 (A) Gene cluster dendrograms and module colors. The gene dendrogram is obtained by overlaying the topology with the corresponding module colors. Each color represents an independent module that contains a set of highly-related genes. (B) Heat map of the correlation between co-expression module genes and clinical features (tumor and normal), the top number in each cell is the correlation coefficient, and the bottom one is the p-value.

The data were processed and analyzed as shown in the flowchart in Fig 1. By filtering the two gene expression matrices, genes in the top 5,000 with absolute median differences in TCGA dataset and GEO dataset were further screened for co-expression network analysis. Soft strengths of β = 3 in TCGA datasets and β = 4 in the GSE66229 datasets were chosen using the function pickSoftThreshold. Then, the co-expression networks were established, and gene modules were identified using the function blockwiseModules. A total of 9 and 14 modules were identified in TCGA datasets and the GSE datasets (Fig 2A), respectively. Each color represents an independent module that contains a set of highly-related genes. Eventually, the relationship between different co-expression modules and clinical features was visualized by heat map (Fig 2B). The top number in each cell is the correlation coefficient, and the bottom one is the p-value. Modules with high correlation with tumor traits and p-value< 0.05were further analyzed, and the results were consistent with one module (pink, with 113 genes) in TCGA datasets, and with two modules (blue, purple, with 1,581 genes in all) in the GEO datasets.

### Analysis of differential gene expression

**Fig 3:**
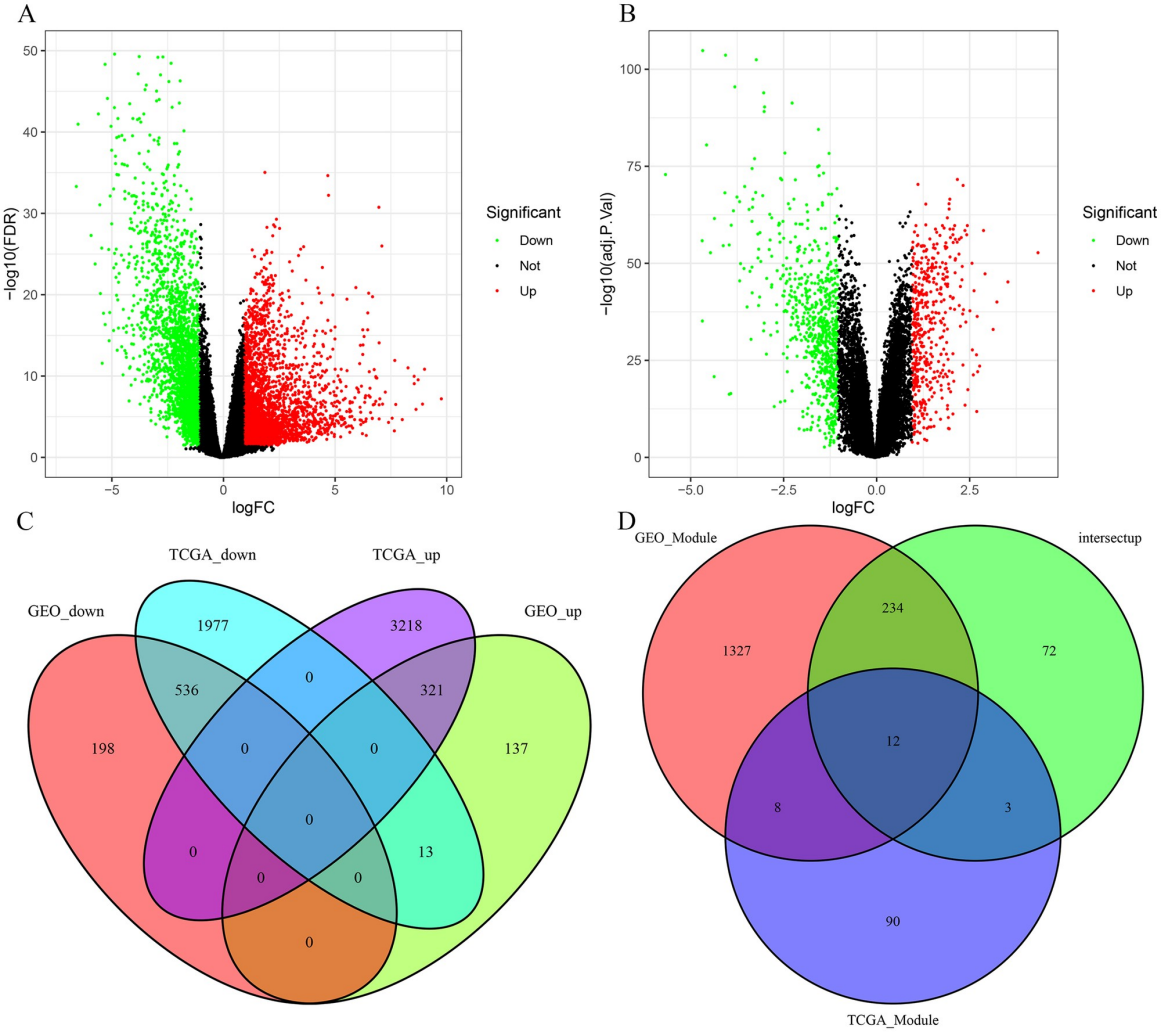
Analysis of differential gene expression with TCGA-GC datasets and the GSE66229 datasets. (A) Volcano plot of TCGA dataset. (B) Volcano plot of the GSE66229 dataset. (C) Venn diagram of genes between DEGs. (D) Venn diagram comparison of genes from the upregulated genes and co-expression modules. A total of 12 overlapping genes were identified.

Using |FC|(fold change)> = 2 and FDR-adjusted P < 0.05 as the cut-off criterion, we obtained 6,065 and 1,205 DEGs from TCGA datasets and the GSE66229 datasets, respectively, and visualized them with volcano plots (Fig 3A and 3B). A total of 857 DEGs (321 upregulated and 536 downregulated) were identified by gene integration analysis (Fig 3C). According to the calculations, there were 12 overlapping genes (*EVA1A*, *RARRES1*, *ADAM12*, *COL10A1*, *COL11A1*, *COL1A1*, *COL1A2*, *COL10A1*, *CTHRC1*, *FAP*, *FNDC1*, *and INHBA*) between the upregulated genes and co-expression Modules (Fig 3D).

### Validation of the actual hub genes

**Fig 4:**
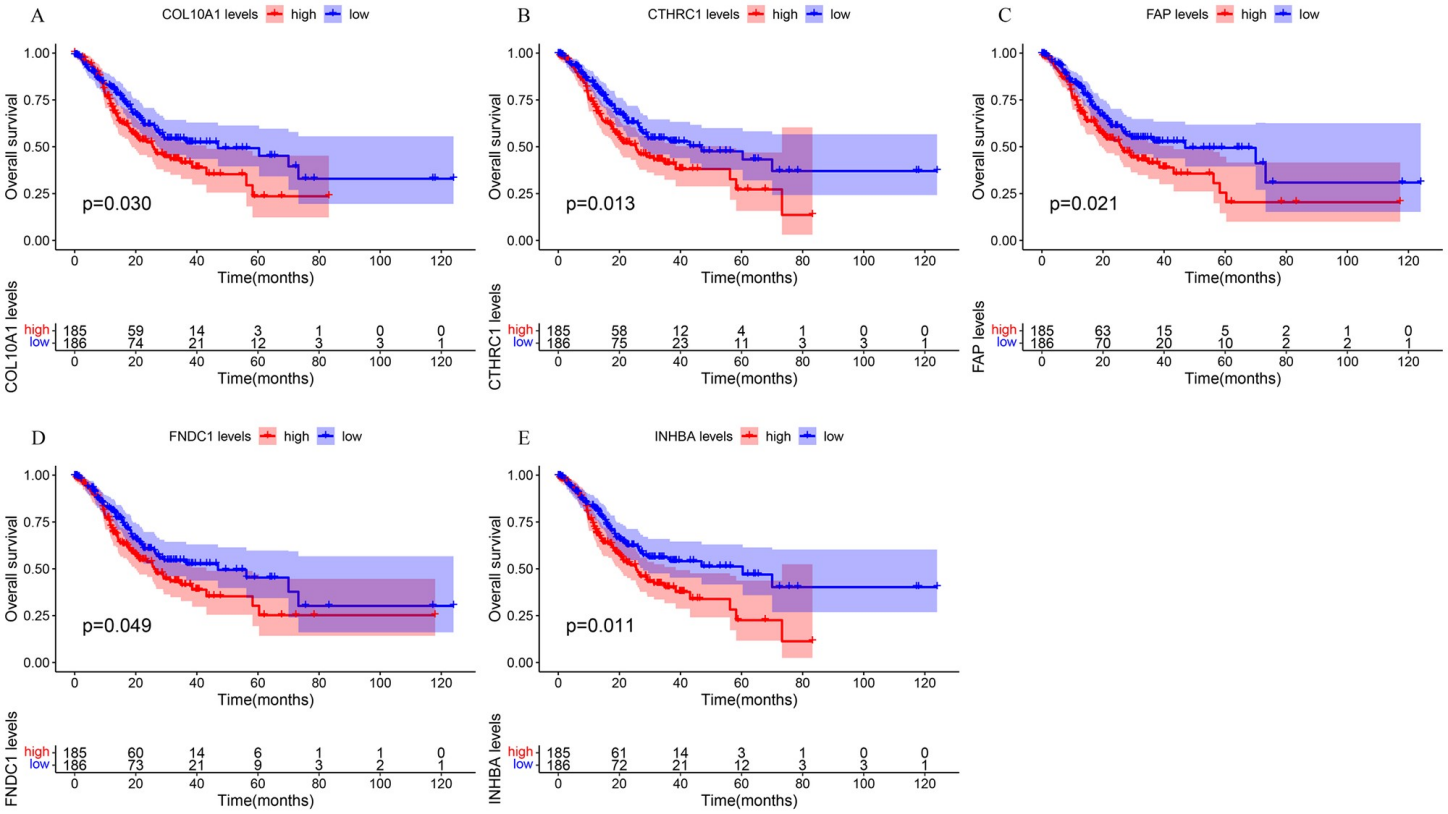
The Kaplan–Meier survival curves of the five genes in TCGA dataset. (A) *COL10A1* (B) *CTHRC1* (C) *FAP* (D) *FNDC1* (E) *INHBA*.

**Fig 5:**
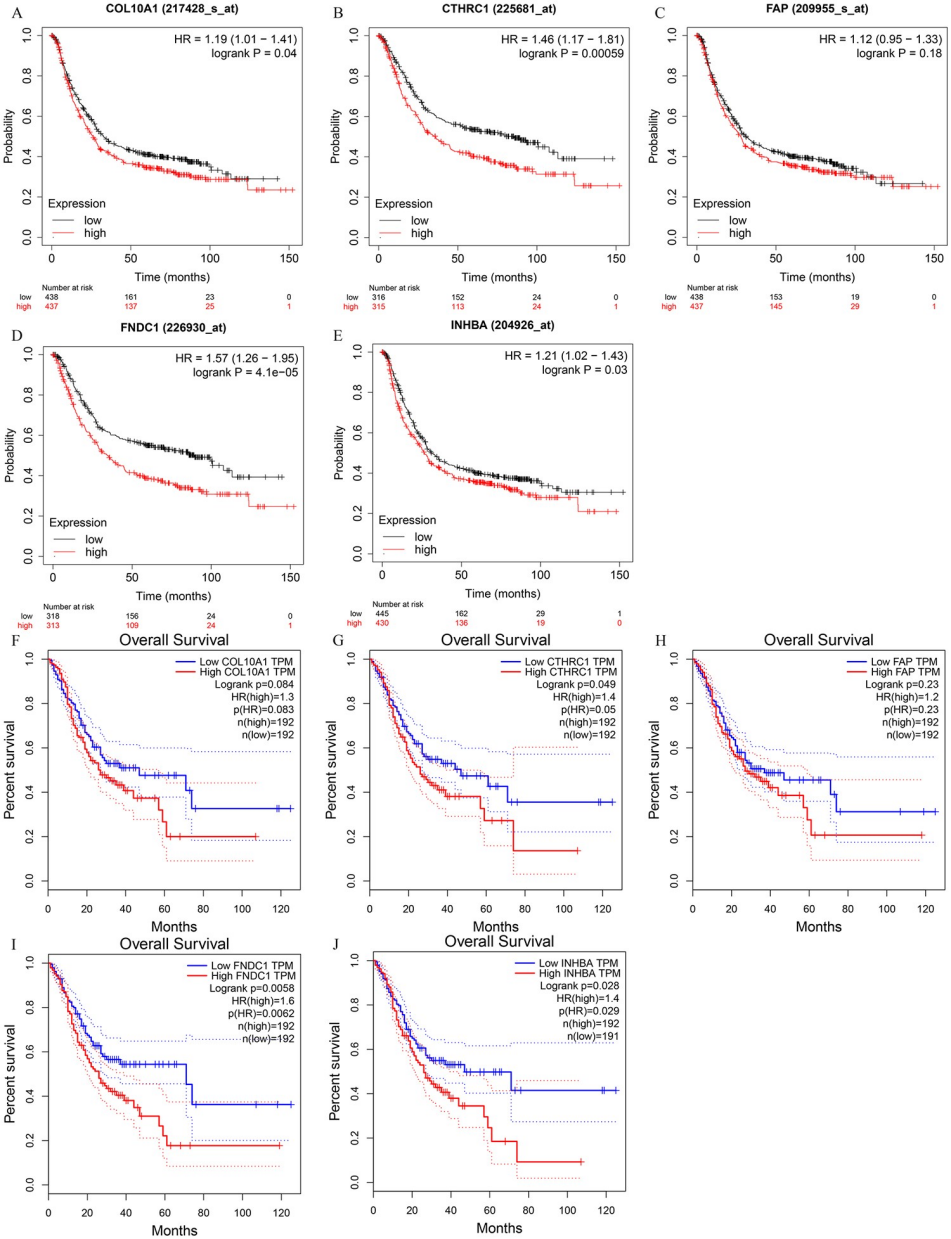
The Kaplan–Meier survival curves of the 5 genes.

**Fig 6:**
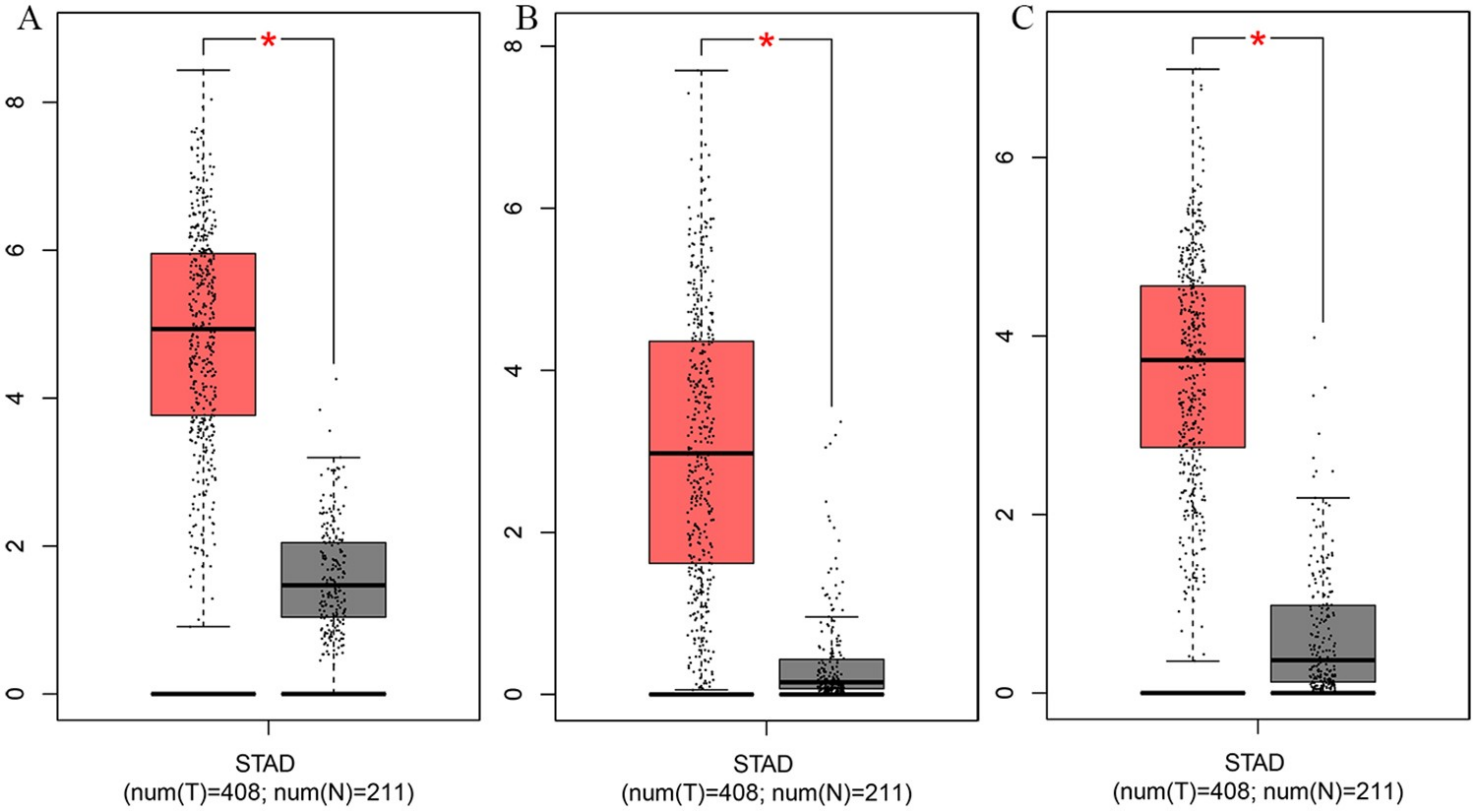
Verification of the expression levels of these 3 hub genes using GEPIA. (A) *CTHRC1* (B) *FNDC1* (C) *INHBA*.

**Fig 7:**
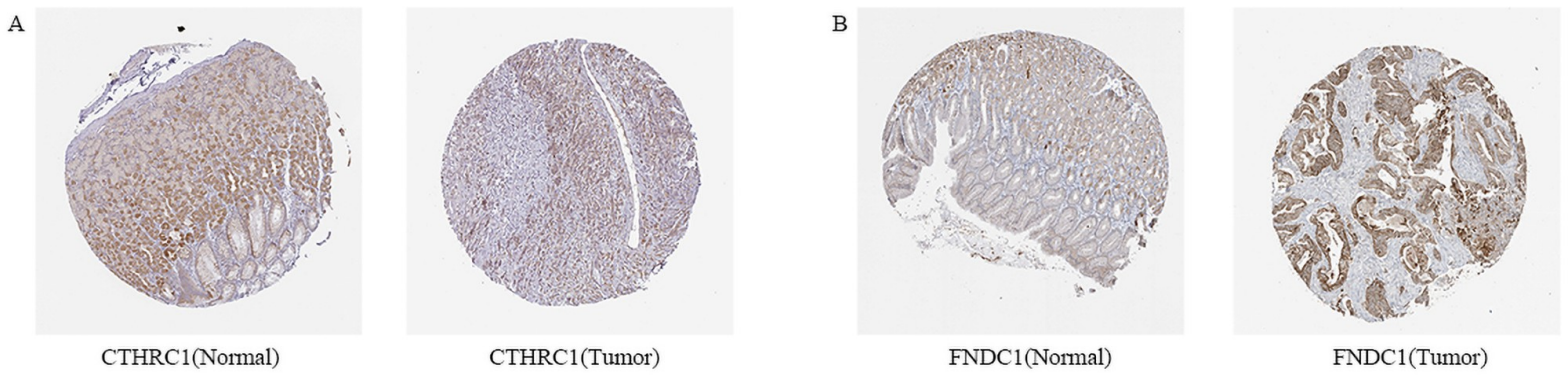
Immunohistochemistry (IHC) analysis of 3 hub genes on the HPA database. (A) *CTHRC1*, (B) *FNDC1*. (*INHBA* was not found in the HPA database).

We identified the prognostic value of the 12 genes in TCGA datasets using the survival package in R. Patients were divided into a high group and a low group based on the median expression of the genes. The results of the Kaplan–Meier curve analysis indicated that the higher the expression of C*OL10A1*, *CTHRC1*, *FAP*, *FNDC1*, *and INHBA*, the worse the prognosis of the GC patients (P < 0.05) (Fig 4). These five genes were further validated by survival analysis using the Kaplan-Meier plotter database (Fig 5A–5E) and GEPIA database (Fig 5F–5J). After the above validation, three candidate genes (*CTHRC1*, *FNDC1*, *and INHBA*) were ultimately determined to be the hub genes. Then, the expression level of these three genes was evaluated using the GEPIA database (Fig 6), which revealed that compared with normal gastric tissue samples, the expression of *CTHRC1*, *FNDC1 and INHBA* was elevated in GC samples. These findings are consistent with the results of our analysis. The IHC staining data obtained from the HPA database were used to determine the protein levels of these three candidate hub genes (Fig 7). The results also showed that the protein levels of *CTHRC1*, *FNDC1* were dysregulated in GC tissues (*INHBA* was not found in the HPA database).

### Gene set enrichment analysis revealed a close relationship between hub genes and tumor development

**Fig 8:**
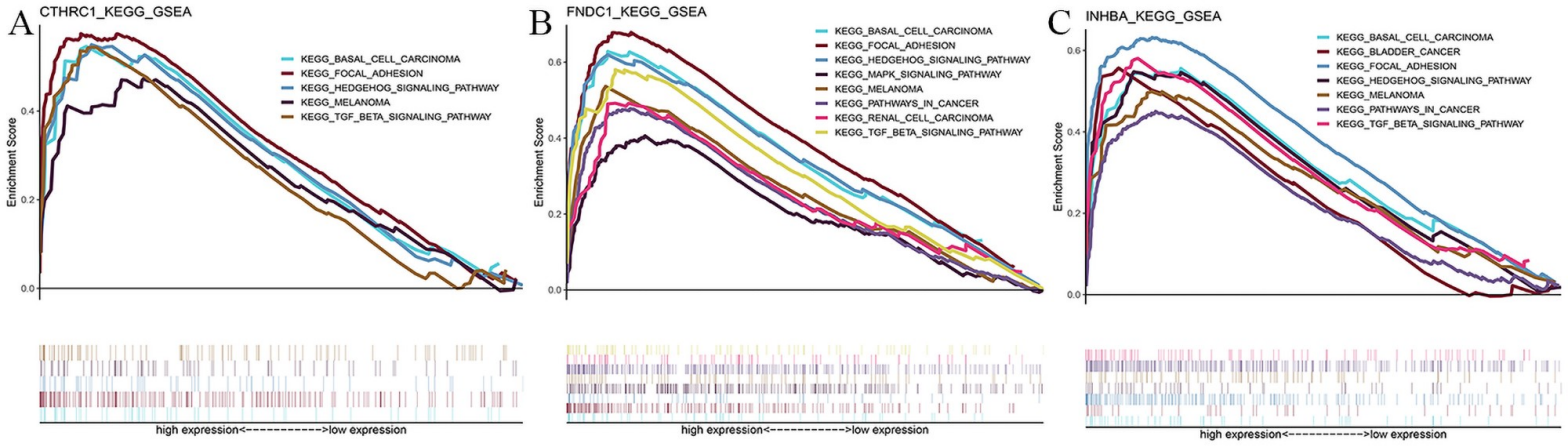
KEGG-GSEA analyses of the 3 identified hub genes. (A) CTHRC1 (B) FNDC1 (C) INHBA.

To further investigate the potential biological functions of *CTHRC1*, *FNDC1*, and *INHBA*, we performed Kyoto Encyclopedia of Genes and Genomes (KEGG)-GSEA analysis of the RNA-seq data of GC samples from TCGA. As shown in Fig 8, genes in higher-expression groups of *CTHRC1*, *FNDC1*, and *INHBA* were all involved in “BASAL CELL CARCINOMA”, “FOCAL ADHESION”, “HEDGEHOG SIGNALING PATHWAY”, “MELANOMA”, and “TGF BETA SIGNALING PATHWAY”. In addition, the “PATHWAY IN CANCER” was enriched in the *FNDC1* and *INHBA* high-expression groups. The sets of genes with the highest enrichment scores are closely related to the occurrence and development of tumor.

### Construction of lncRNA-miRNA–hub gene network

**Fig 9:**
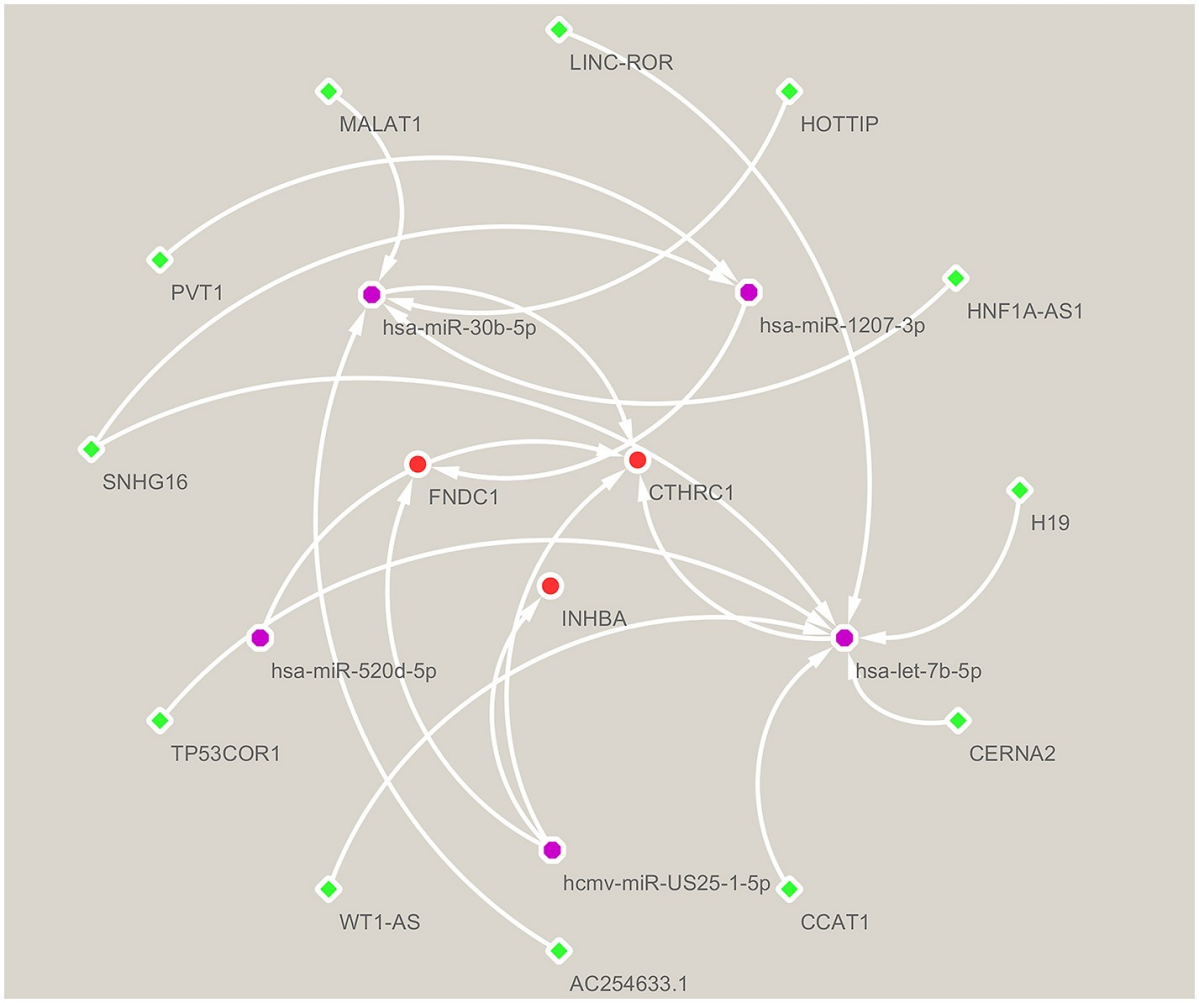
The lncRNA-miRNA-mRNA network was established using the cytoscape software.

We also undertook to establish the transcriptional regulatory network of lncRNAs, miRNAs, and hub genes by selecting strong experimental evidence in the RNAInter database. As depicted in Fig 9, the network includes 12 lncRNAs, 5 miRNAs, and 3 hub genes. The scores of the correlations between lncRNA and miRNA and between miRNA and mRNA are shown in Table 1. MiRNAs regulate gene expression by interacting with their target genes [22]. LncRNAs function as competing endogenous RNAs (ceRNAs), competing for shared miRNAs and sequestering miRNAs from mRNAs [23]. This network reflects the regulatory relationships in the process of hub genes expression as well as the complex mechanisms of tumorigenesis.

**Table 1:** The correlation between lncRNA, miRNA, and mRNA according to the RNAInter database.

RNAInter_ID	Interactor1	Category1	Interactor2	Category2	Score
RR00377685	hsa-let-7b-5p	miRNA	CTHRC1	mRNA	0.9943
RR00377701	hsa-miR-30b-5p	miRNA	CTHRC1	mRNA	0.9928
RR00377703	hsa-miR-520d-5p	miRNA	CTHRC1	mRNA	0.9609
RR00377694	hcmv-miR-US25-1-5p	miRNA	CTHRC1	mRNA	0.7311
RR00572052	hsa-miR-1207-3p	miRNA	FNDC1	mRNA	0.9526
RR00572047	hcmv-miR-US25-1-5p	miRNA	FNDC1	mRNA	0.7311
RR00751831	hcmv-miR-US25-1-5p	miRNA	INHBA	mRNA	0.7311
RR00694900	H19	lncRNA	hsa-let-7b-5p	miRNA	1
RR01338124	SNHG16	lncRNA	hsa-let-7b-5p	miRNA	0.9856
RR00287286	CCAT1	lncRNA	hsa-let-7b-5p	miRNA	0.982
RR01472766	TP53COR1	lncRNA	hsa-let-7b-5p	miRNA	0.9526
RR00845074	LINC-ROR	lncRNA	hsa-let-7b-5p	miRNA	0.9526
RR00320272	CERNA2	lncRNA	hsa-let-7b-5p	miRNA	0.9526
RR05191717	WT1-AS	lncRNA	hsa-let-7b-5p	miRNA	0.7704
RR00719481	HOTTIP	lncRNA	hsa-miR-30b-5p	miRNA	0.9975
RR00032111	AC254633.1	lncRNA	hsa-miR-30b-5p	miRNA	0.9912
RR00715424	HNF1A-AS1	lncRNA	hsa-miR-30b-5p	miRNA	0.982
RR00889541	MALAT1	lncRNA	hsa-miR-30b-5p	miRNA	0.7311
RR01338152	SNHG16	lncRNA	hsa-miR-1207-3p	miRNA	0.9818
RR01159638	PVT1	lncRNA	hsa-miR-1207-3p	miRNA	0.8808

## Discussion

In this study, we first identified tumor-associated co-expression modules in two datasets using WGCNA, and then the DEGs between tumor and normal tissues, ultimately identifying a total of 12 overlapping genes between the upregulated genes and co-expression modules. These genes were not only upregulated in GC but also highly associated with GC. We additionally performed a prognostic analysis of these genes using TCGA database and further validated them using the Kaplan-Meier plotter database and GEPIA database. The GEPIA and Kaplan-Meier plotter databases are two web-based tools that deliver fast and customizable functionalities. The GEPIA database provides key interactive and customizable functions including profiling plotting, differential expression analysis, patient survival analysis base on TCGA and GTEx data [24]. The Kaplan-Meier Plotter database includes gene expression data and clinical data, which is a powerful tool that can be used to evaluate the effect of genes on the survival of patients with gastric cancer [25]. The use of these two databases can make our results more reliable and compelling. After the validation process, revealed that higher expression of *CTHRC1*, *FNDC1*, and *INHBA* indicated poorer survival in patients with GC, these 3 genes were considered to be the hub genes. The KEGG-GSEA analysis indicated that these mRNAs were significantly enriched in cancer-related pathways, including basal cell carcinoma, focal adhesion [26], hedgehog signaling pathway [27], melanoma, pathway in cancer, and TGF beta signaling pathway [28].

*CTHRC1* (Collagen Triple Helix Repeat Containing 1) is a protein-encoding gene that appears to play a role in the cellular response to arterial injury through its involvement in vascular remodeling. It has been reported that, following injury, *CTHRC1* is transiently overexpressed in the adventitial and intimal smooth muscle of rat arteries [29]. Although physiologically *CTHRC1* plays an important role in wound healing, abnormal expression of *CTHRC1* also promotes the development of various human tumors. For instance, *CTHRC1* promotes cervical cancer metastasis and activates the Wnt/PCP pathway [30]. In addition, *CTHRC1* induces non-small cell lung cancer invasion by upregulating MMP-7/MMP-9 [31]. Moreover, in gastric cancer, *CTHRC1* was reported to promote tumor metastasis through the HIF-1α/CXCR4 signaling pathway [32]. *FNDC1* encodes a protein containing a fibronectin type III structural domain. Fibronectin interaction with integrins is involved in cell proliferation, migration, and differentiation [33]. Recent studies have found that *FNDC1* also has a role in different diseases including cancer. *FNDC1* was shown to be involved in the pathological changes in inflammatory bowel disease [34]. The silencing of *FNDC1* inhibited the proliferation and migration of prostate cancer cells [35]. *FNDC1* was also shown to promote apoptosis through hypermethylation in human salivary-like cystic carcinoma cells [36]. Additionally, high *FNDC1* expression was also reported to be associated with poor prognosis in gastric cancer [37], which is consistent with our findings. *INHBA* encodes a member of the TGF-beta superfamily of proteins, which has been found to be associated with various types of human cancers. Previous studies have shown that besides being associated with cell proliferation and migration, the *INHBA* gene is overexpressed in various tumors, such as colorectal cancer [38], esophageal cancer [39], and nasopharyngeal cancer [40]. All the above studies similarly indicate that these three genes may serve as potential diagnostic and prognostic biomarkers for gastric cancer. However, few studies have investigated the important upstream mediators of these hub genes. In this study, we made predictions about the upstream regulatory mechanisms of these genes using the RNAinter database in combination with the strong experimental evidence and established a regulatory network of lncRNAs-miRNAs-hub genes of GC, involving 12 lncRNAs, 5 miRNAs, and 3 hub genes, such as models HOTTIP-miR30b-*CTHRC1*, H19-let7b-*CTHRC1*, and PVT1-miR1207-*FNDC1*.

Non-coding RNAs, such as lncRNAs, circRNAs, and miRNAs, which were considered as transcriptional noise in the past and are now known to account for over 90% of the human genome [41], have been shown to have regulatory roles in various biological processes and play a crucial role in the development of diseases [42]. In recent years, considerable attention has been devoted to some calculation methods for predicting the potential associations of miRNAs, lncRNAs, circRNAs, and diseases as they can provide the most promising reference for the experiment, greatly reducing the time and cost of the experiment [43–45]. For instance, Chen *et al*. proposed Matrix Decomposition and Heterogeneous Graph Inference for miRNA-disease association prediction (MDHGI) by combining the sparse learning method with the heterogeneous graph inference method to calculate and predict the association of potential miRNA and disease [46]. Additionally, Chen *et al*. also developed a model of Inductive Matrix Completion for MiRNA-Disease Association prediction (IMCMDA), which was successfully validated in five human tumors [47]. Advances in interaction prediction research in various fields of computational biology have also provided valuable insights for the development of mRNA-miRNA-lncRNA networks, such as miRNA-lncRNA interaction prediction. Zhang *et al*. constructed the LMI-INGI and NDALMA models to predict the interactions between lncRNAs and miRNAs, and obtained satisfactory results in five-fold cross-validation, showing good prediction performance [48, 49]. LMFNRLMI is another algorithm which can achieve a good prediction of the relationship between lncRNA and miRNA [50]. Liu et al. proposed the "IMBDANET" algorithm that can predict genes directly or indirectly related to the target gene [51]. In the future, we can try to use these computational models to identify non-coding RNA biomarkers for gastric cancer and explore potential regulatory networks.

However, this study has several limitations, including the following. First, some key genes may have been removed during the gene filtering performed before performing WGCNA analysis. Second, when analyzing DEGs, some factors were not considered, such as age, sex, tumor staging, and patient classification. Finally, although the upstream regulatory network of the three hub genes has been predicted, experiments are still needed for further verification.

## Conclusions

In conclusion, through comprehensive bioinformatics analysis, we successfully identified three hub genes (*CTHRC1*, *FNDC1*, and *INHBA*) that are differentially expressed between tumor and normal tissues in both TCGA-STAD and GSE66229 datasets and highly correlated with GC. These genes may play key roles in the development of gastric cancer. Their upstream lncRNA and miRNA regulators may reveal the potential mechanism by which these hub genes modulate the progression of GC. Overall, this study provides a new perspective on the diagnosis, prognosis, and treatment strategies for this malignant disease.

## Supporting information

S1 Checklist (PDF)

S2 Checklist (DOCX)
